# Agricultural Commodity Price Volatility and Adolescent Reproductive Health in Developing Economies: Pathways, Controversies, and Policy Priorities

**DOI:** 10.3390/ijerph23070851

**Published:** 2026-06-30

**Authors:** Ángel Maridueña-Larrea, Washington Guevara-Piedra, Marco Faytong-Haro, Javier Chiliquinga-Amaya, Rocio Gonzalez-Reyes, Patricio Alvarez-Muñoz

**Affiliations:** 1 Facultad de Ciencias Sociales, Educación Comercial y Derecho, Universidad Estatal de Milagro, Milagro 091050, Ecuador; amariduenal@unemi.edu.ec (Á.M.-L.); wguevarap@unemi.edu.ec (W.G.-P.); jchiliquingaa@unemi.edu.ec (J.C.-A.); palvarezm@unemi.edu.ec (P.A.-M.); 2 Facultad de Investigación, Universidad Estatal de Milagro (UNEMI), Milagro 091050, Ecuador; 3 Facultad de Vinculación, Universidad Estatal de Milagro (UNEMI), Milagro 091050, Ecuador; rgonzalezr2@unemi.edu.ec; 4 Facultad de Ciencias Económicas, Universidad de Guayaquil, Guayaquil 090506, Ecuador

**Keywords:** commodity price volatility, adolescents, reproductive health, developing economies, food insecurity

## Abstract

**Highlights:**

**Public health relevance—How does this work relate to a public health issue?**

- This narrative review synthesizes evidence on how agricultural commodity price volatility shapes upstream determinants of adolescent sexual and reproductive health, including food security, schooling, and access to care.
- It shows that economic shocks in commodity-dependent settings increase vulnerability to early marriage, unintended pregnancy, and unsafe reproductive outcomes, especially among rural girls.

**Public health significance—Why is this work of significance to public health?**

- By integrating findings across disciplines, this narrative review reframes market instability as a structural determinant of adolescent reproductive health beyond proximate behavioral factors.
- It identifies consistent indirect pathways linking economic shocks to adverse health outcomes and highlights the lack of age-disaggregated causal evidence.

**Public health implications—What are the key implications or messages for practitioners, policy makers and/or researchers in public health?**

- Findings support integrated policy responses that connect food systems, social protection, education, and adolescent-friendly health services in shock-prone regions.
- The review underscores the need for early warning systems based on commodity prices to trigger targeted interventions that protect adolescent well-being during economic downturns.

**Abstract:**

Agricultural commodity markets remain central to household survival across many developing economies, yet their volatility is rarely framed as an adolescent sexual and reproductive health problem. This mini review uses a structured narrative approach anchored in a screened evidence map of 1065 records, from which 50 papers were retained and 16 studies were prioritized for full synthesis. We define adolescent reproductive health holistically, including sexual agency, contraceptive information and use, pregnancy intention, antenatal and obstetric care, protection from coercion, and maternal and neonatal outcomes. The review provides a concrete answer to the primary question: agricultural commodity price volatility is a distal, context-conditioned determinant of adolescent reproductive health, not a uniform direct cause. Its effects operate mainly through food security, household income, labor allocation, school continuity, gendered bargaining power, and service access. Negative shocks more consistently erode nutrition, schooling, transport to care, and access to adolescent-friendly services, especially among rural girls in households with weak shock buffers. Positive shocks may increase births or union formation when income effects dominate, but they may also harm health when higher labor demand raises the opportunity cost of caregiving and service use. Direct adolescent-specific causal evidence remains limited; therefore, adjacent evidence on fertility, child health, schooling, and maternal or neonatal outcomes is interpreted through an explicit evidence hierarchy rather than treated as equivalent to direct adolescent evidence. Policy priorities include shock-responsive social protection, school retention, contraceptive supply continuity, adolescent-friendly care, and early warning systems that trigger health and education responses during commodity instability.

## 1. Introduction

In many low- and middle-income countries, agricultural commodity prices do more than shape export earnings. They alter wages, labor demand, food affordability, caregiving time, school participation, and the capacity of households to absorb shocks. For adolescents, these shifts matter because sexual and reproductive health is tightly tied to the material conditions of daily life. In this review, adolescents refer to persons aged 10–19 years, and adolescent reproductive health is used in a holistic sexual and reproductive health sense: physical, mental, and social well-being in relation to sexuality and reproduction; access to information, contraception, sexually transmitted infection prevention, pregnancy-related care, and safe services where legally available; freedom from coercion and violence; and the ability to make informed reproductive decisions [1,2].

The conceptual argument draws on social determinants and life-course approaches to adolescent health [3,4]. Commodity price volatility is treated as a distal structural exposure that becomes relevant for adolescent reproductive health only after it is translated through household resources, gendered labor, school attachment, service availability, and local norms. This approach avoids reducing adolescent reproductive health to fertility alone and recognizes maternal and neonatal endpoints as part of the same continuum of reproductive vulnerability.

Developing economies and commodity-dependent settings are not assumed to be homogeneous. The term is used here for contexts that commonly share several features relevant to this review: high dependence on agricultural production or food markets for livelihoods, large food-expenditure shares among poor households, weaker social protection, rural service gaps, and gendered constraints on adolescent autonomy. The magnitude and direction of any shock can differ sharply between net food consumers, landholding producers, wage-labor households, and regions with different health-system capacity. Therefore, the synthesis treats a coffee price shock in a middle-income setting, a cocoa price collapse in a low-income setting, and a staple-food price spike as comparable only at the level of pathways, not as identical empirical events.

Despite this logic, the literature linking agricultural commodity volatility directly to adolescent reproductive health remains thin. Most high-quality studies examine total fertility, child nutrition, mortality, birth outcomes, or educational attainment rather than adolescent pregnancy, contraception, or pregnancy intention. These adjacent outcomes are not used as substitutes for direct evidence. Instead, they are used to identify plausible mechanisms and to specify where direct adolescent-focused research is still needed.

This mini review argues that agricultural commodity price volatility should be understood as a distal but policy-relevant determinant of adolescent reproductive health. The strongest evidence concerns three interlocking pathways: household income and fertility responses, nutrition and schooling, and service access within unequal gender regimes. The review also addresses a central controversy in the field: whether income and consumption losses dominate, or whether changes in the opportunity cost of caregiving time can produce different effects. For adolescent outcomes, the implication is that price shocks cannot be treated as uniformly beneficial or harmful; their effects depend on labor markets, health systems, schools, and preexisting inequality.

## 2. Review Approach and Article Selection

This manuscript used a structured narrative review approach rather than a formal systematic review or meta-analysis. The evidence base was constructed from a structured search of peer-reviewed literature conducted during March 2026, drawing on studies indexed in major academic databases, including PubMed, Scopus, Web of Science, CrossRef, and Semantic Scholar. Search strings combined terms related to agricultural commodity prices (for example, food prices, commodity price volatility, agricultural shocks, export-crop shocks), adolescent populations (adolescents, youth, teenagers), and reproductive health outcomes or pathways (fertility, early pregnancy, sexual and reproductive health, contraception, maternal health, child health, nutrition, schooling, and service access). Boolean operators and term combinations were refined iteratively to maximize coverage across economics, demography, public health, and development studies.

**Figure 1:**
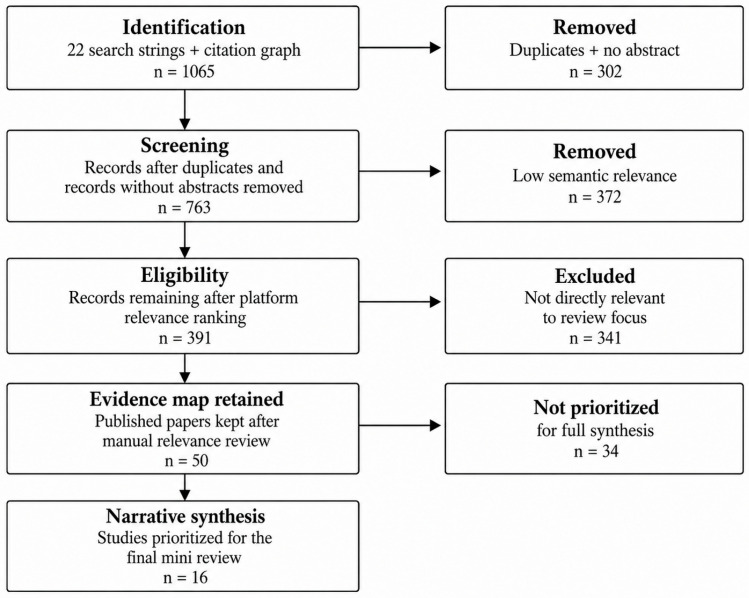
Screening and prioritization flow for the structured narrative mini review.

The search strategy yielded 1065 records. After removal of duplicates and records without abstracts, 763 records remained for title and abstract screening. Platform relevance ranking excluded 372 low-relevance records, leaving 391 records for manual assessment. From these, 50 published papers were retained in the evidence map, and 16 were prioritized for narrative synthesis because they offered the strongest conceptual or empirical leverage for the review question (Figure 1). The final set of 16 papers should not be interpreted as a 1.5% “retention rate” meant to represent all evidence returned by the initial search. It is the deliberately focused synthesis subset drawn from a broader evidence map after relevance screening.

Manual screening prioritized peer-reviewed empirical studies and systematic reviews conducted in low- and middle-income countries, sub-Saharan Africa, upper-middle-income settings with persistent adolescent reproductive vulnerability, or other commodity-dependent contexts relevant to the mechanisms under review. Studies were retained when they examined at least one of the following: agricultural commodity or food price shocks; fertility, maternal or child health, nutrition, schooling, or birth outcomes plausibly linked to adolescent reproductive risk; or adolescent sexual and reproductive health determinants and interventions. Studies were deprioritized when they focused on high-income settings without clear transferability, unrelated macroeconomic events, methods without substantive findings, or outcomes with no plausible connection to reproductive health.

To reduce speculative extrapolation, the final synthesis used an explicit evidence hierarchy. Direct evidence refers to studies with adolescent sexual and reproductive health outcomes, such as adolescent pregnancy, contraception, or adolescent service use. Pathway evidence refers to studies of fertility, child health, nutrition, birth outcomes, schooling, or labor allocation that identify mechanisms plausibly affecting adolescents but do not directly measure adolescent reproductive outcomes. Contextual evidence refers to health-system or intervention studies that clarify whether services can buffer risk. Conclusions are strongest when direct and pathway evidence align, and more cautious when only proxy evidence is available.

A pooled quantitative estimate was not attempted because the available studies differed substantially in exposure definition, commodity type, country income level, household position in the commodity economy, outcome measurement, age aggregation, and empirical design. Synthesizing these data as if they estimated a common effect would risk false precision. The review therefore provides a concrete qualitative answer: commodity volatility is most likely to harm adolescent reproductive health when it simultaneously worsens food affordability, reduces school attachment, increases economic dependence on partners or labor markets, and weakens access to adolescent-friendly care. The manual screening and prioritization criteria applied during study selection are summarized in Table 1.

## 3. Commodity Shocks, Household Welfare, and Fertility Responses

The most direct evidence connecting commodity markets to reproductive behavior comes from studies of fertility responses to local economic shocks. In a quasi-experimental analysis from Chile, Gallego and Lafortune found that international commodity booms increased births and birth rates, with effects concentrated in higher-order births rather than family initiation. They also found fewer conceptions among single women and more favorable partner characteristics during booms, suggesting that commodity upswings may reshape family formation rather than simply increase exposure to pregnancy [5].

For adolescent health, aggregate fertility is an imperfect indicator. A rise in total births may reflect planned parity progression among adult women, whereas adolescent pregnancies may move in a different direction. Positive shocks can relax liquidity constraints, reduce immediate economic distress, and make it easier for families to keep girls in school. At the same time, higher local incomes can make earlier union formation more feasible and can normalize larger family size if gains are concentrated among households already embedded in pronatalist settings. Conversely, downturns may suppress intended fertility at the aggregate level while increasing unintended pregnancy among disadvantaged adolescents if school exit, transactional sex, or disrupted contraceptive use intensify.

This distinction explains why the fertility literature and the adolescent pregnancy literature only partially overlap. Reviews of adolescent pregnancy in Africa and other low- and middle-income settings consistently identify poverty, low educational attainment, rural residence, age-disparate partnerships, and constrained reproductive agency as major drivers of early pregnancy [6,7,8]. More recent cohort evidence from Southeastern Europe similarly shows that adolescent pregnancy can remain concentrated among rural and low-education populations even in upper-middle-income settings, with adverse obstetric and neonatal endpoints linked to structural disadvantage and health-system access [9]. Although this cohort evidence is not commodity-specific, it strengthens the external validity of the conceptual model by showing that adolescent reproductive vulnerability is shaped less by national income category alone than by socioeconomic inequality, rural access barriers, educational discontinuity, and system resilience.

## 4. Nutrition, Schooling, and the Life-Course Production of Reproductive Risk

The strongest evidence from developing economies points to indirect pathways running through nutrition, child health, and schooling. During the 1990 cocoa crisis in Côte d’Ivoire, commodity price collapse was associated with worse child outcomes in cocoa-growing areas, including deteriorating school and health conditions that undermined human capital formation [10]. This historical evidence is retained as mechanism evidence rather than as a direct forecast for the 2026 service environment. Digital health access, expanded contraceptive distribution, and more diversified service-delivery models can moderate the consequences of shocks, but they do not remove the relevance of cash constraints, transport costs, confidentiality concerns, commodity stockouts, or school exit in rural settings.

Several studies show how food and commodity price movements become biologically embedded early in life. Cross-national work linked commodity price volatility to higher child mortality in low- and middle-income countries, reinforcing the view that price instability can damage child well-being where social protection is weak and dependence on primary commodities is high [11]. In Tanzania, commodity price fluctuations were associated with child malnutrition, underscoring the long-run consequences of shocks experienced around birth and early childhood [12]. Across 44 developing countries, even modest increases in real food prices raised the risk of wasting and severe wasting among preschool children [13]. In Kenya, maize price dynamics were linked to birth weight, showing that food-market conditions can affect reproductive health even before a child is born [14].

These findings matter for adolescent reproductive health because early nutritional insult and educational disruption accumulate over time. Adolescents who enter puberty after chronic undernutrition, interrupted schooling, or repeated household stress face weaker bargaining power, lower perceived returns to continued education, and greater vulnerability to early union and pregnancy. Evidence from Malawi is especially instructive: girls exposed to multiple negative economic shocks around birth had lower cognitive scores and lower educational attainment in adolescence, whereas comparable effects were not observed among boys [15]. Once schooling trajectories diverge, so do opportunities to delay marriage, negotiate contraceptive use, and avoid economically coercive sexual relationships.

At the same time, the health effects of commodity shocks are not uniformly countercyclical. In Colombia, coffee price increases were associated with procyclical child mortality in coffee-growing regions because adults worked more and time-intensive health investments fell [16]. This finding shifts attention from household income to the opportunity cost of caregiving time. For adolescents, the same logic implies that a boom can improve cash income while worsening supervision, school attendance, service use, or autonomy if girls or caregivers are pulled into labor. The direction of effect therefore depends on whether the dominant channel is income and consumption, food affordability, or time allocation.

## 5. Gendered Service Access and Crisis-Mediated Vulnerability

Adolescent reproductive outcomes are produced within unequal social systems, so commodity volatility is filtered through gender, geography, household bargaining, and service access. Reviews from sub-Saharan Africa and other low- and middle-income settings show that early pregnancy is concentrated among girls with low schooling, weak family support, rural residence, poverty exposure, and limited autonomy in sexual decision-making [6,7,8]. These are precisely the conditions most likely to intensify during commodity downturns, particularly in regions where households depend on a narrow set of crops and have limited capacity to smooth consumption.

Access to contraception and abortion-related knowledge is also uneven long before a shock occurs. Adolescents in low- and middle-income countries face persistent barriers to contraception knowledge, access, and use [17], and global progress in adolescent sexual and reproductive health has been highly unequal across and within countries [2]. Against this background, a price bust does not need to create a new barrier to harm adolescent health; it only needs to deepen existing ones. Reduced cash for transport, greater dependence on male partners, interruptions in schooling, and pressure to contribute labor or income can all narrow the practical ability of adolescents to obtain information, negotiate condom use, continue antenatal care, or use services confidentially.

The intervention literature suggests that these risks are modifiable. Comprehensive sexuality education, adolescent-friendly health services, and multicomponent community strategies can improve key sexual and reproductive health outcomes [18]. Evidence from humanitarian settings shows that adolescents are often overlooked during crises even though targeted, youth-responsive sexual and reproductive health programming is feasible and necessary [19]. Commodity downturns are not identical to armed conflict or displacement, but they can generate comparable patterns of service fragility and household desperation in commodity-dependent areas. Recent clinical evidence on maternal and neonatal outcomes in organized delivery models further illustrates that reproductive outcomes are shaped not only by exposure to risk, but also by the organization, accessibility, and continuity of care available when risk occurs [20]. This health-system perspective broadens the policy focus from fertility prevention alone to antenatal access, safe delivery, neonatal outcomes, and postpartum continuity.

## 6. Competing Interpretations and Current Controversies

Two controversies structure the field. The first concerns mechanism. A useful adjudication framework asks four questions: whether the household is a net producer, wage laborer, or net food consumer; whether the shock primarily changes cash income, food affordability, or labor demand; whether women and adolescent girls are pulled into paid or unpaid labor; and whether schools and health services can buffer the shock. The income and consumption mechanism is more likely to dominate when households are net food buyers or landless laborers, food-expenditure shares are high, social protection is weak, and service costs or transport expenses are binding. The opportunity-cost mechanism is more likely to dominate during commodity booms that raise demand for adult female labor, adolescent labor, or caregiver time, especially when preventive care and school attendance require time-intensive investments. This framework helps explain why evidence on food prices, malnutrition, and child mortality points to harm from price increases or volatility [11,12,13], whereas evidence from coffee-producing Colombia shows harm during booms through time-allocation channels [16].

The second controversy concerns scale of inference. Direct evidence is currently strongest for adolescent pregnancy determinants and interventions, but weakest for causal estimates linking local commodity exposure to adolescent contraception, pregnancy intention, unsafe abortion, sexually transmitted infections, or service use. Proxy evidence is most reliable when it concerns pathways that are closely connected to adolescent reproductive risk, such as school discontinuity, food insecurity, transport barriers, and household labor allocation. It is more speculative when it attempts to infer adolescent pregnancy intention or contraceptive autonomy from total fertility or child health outcomes alone. The main risks of extrapolation are age aggregation, ecological fallacy, unmeasured climate or policy shocks, and the assumption that household-level effects are evenly distributed across girls and boys. The synthesis therefore treats adjacent evidence as pathway evidence and not as proof of a direct adolescent causal effect.

## 7. Discussion

The primary research question can be answered as follows: agricultural commodity price volatility is a recurring upstream determinant of adolescent reproductive health, but its effects are conditional rather than uniform. The most defensible conclusion is not that every price shock directly increases adolescent pregnancy. Rather, volatility increases adolescent reproductive risk when it undermines food security, weakens school continuity, increases dependence on partners or labor markets, and reduces timely access to confidential, adolescent-friendly care. The evidence is strongest for negative shocks, which repeatedly appear alongside undernutrition, mortality, educational setbacks, and other forms of adversity that elevate adolescent reproductive vulnerability. Rural girls in commodity-dependent households appear especially vulnerable because they are more exposed to income loss, more likely to leave school under stress, and less able to compensate for weak service systems.

A practical implication follows for policy design. Ministries of health and agriculture often treat commodity volatility as a macroeconomic or food-systems problem, while adolescent sexual and reproductive health is managed within a separate program silo. The evidence reviewed here suggests that this separation is costly. When a major export crop’s price collapses or staple prices spike, the policy response should not focus only on producer support or market stabilization. It should also protect the social conditions that allow adolescents, especially girls, to delay pregnancy safely and to receive care when pregnancy occurs: school attendance, food security, transport to care, confidential contraception, antenatal care, safe delivery, postpartum follow-up, and protection from coercion and violence. In commodity-dependent districts, early warning systems based on price and climate indicators could be tied to temporary cash transfers, school meal expansion, mobile or school-linked outreach, digital follow-up where feasible, community health worker visits, and replenishment of reproductive health commodities before households shift into crisis coping.

At the same time, positive shocks should not be romanticized. Booms can raise births, expand family size, and pull women and older girls into labor markets in ways that do not necessarily improve reproductive autonomy. The key question is not whether a shock is positive or negative in the aggregate, but whether it strengthens or weakens the capability of adolescents to stay in school, avoid coercive partnerships, obtain contraception, access antenatal and delivery care, and continue care after birth.

Current research gaps remain substantial. First, there is little causal evidence that directly links local agricultural price exposure to adolescent pregnancy, contraceptive behavior, unsafe abortion, sexually transmitted infection risk, or adolescent-specific maternal and neonatal outcomes. Second, the literature rarely distinguishes between landowning farmers, wage-labor households, landless consumers, and adolescents whose labor or schooling responds differently to the same price movement. Third, few studies integrate commodity exposure with service-side indicators such as contraceptive stockouts, clinic utilization, transport costs, school attendance, adolescent-friendly service quality, or delivery-care capacity. Fourth, adolescent boys are often missing from the analysis except as labor-market actors, limiting understanding of how masculinities, partnership dynamics, and male migration mediate risk for girls.

Potential future developments in the field are promising. Better linkage of geocoded household surveys, market price series, climate data, school records, and health facility data could identify whether shocks alter adolescent outcomes at specific developmental windows. Longitudinal cohorts are especially needed to test whether early-life commodity shocks influence puberty timing, school completion, first union, first birth, antenatal care, and neonatal outcomes. On the policy side, shock-responsive cash transfers, school feeding, fee waivers, contraceptive supply protection, adolescent-focused outreach, and continuity-of-care models deserve rigorous evaluation in commodity-dependent districts. Recent evidence on maternal and child health under economic growth and recession in sub-Saharan Africa likewise suggests that institutions matter greatly [21]. That insight should be extended to adolescents: strong health systems and social protection do not eliminate commodity shocks, but they can prevent those shocks from becoming reproductive crises.

Agricultural commodity volatility should therefore be treated not as background economic noise, but as a recurring social determinant of adolescent reproductive health. A more explicit integration of demography, public health, health-systems research, and development economics would make the field better able to identify who is harmed, through which mechanisms, and which interventions can protect adolescents when markets turn against them.

## 8. Conclusions

The reviewed literature suggests that agricultural commodity price volatility is best understood as a structural context that can intensify adolescent reproductive vulnerability where household insecurity, gender inequality, and weak service systems already intersect. Its influence is not uniform across settings and cannot be reduced to a simple positive or negative shock effect. The broader significance of this evidence lies in shifting attention from individual reproductive behavior alone toward the economic and institutional conditions that shape adolescent choices and constraints. Future studies should prioritize age-disaggregated causal designs that link local market shocks with adolescent pregnancy, contraceptive access, schooling, and service-use outcomes.

## Figures and Tables

**Table 1:** Manual screening and prioritization criteria.

Criterion	Included	Excluded or Deprioritized
Population and setting	Low- and middle-income countries, sub-Saharan Africa, or other commodity-dependent settings relevant to developing economies.	High-income or historical settings without clear transferability to present developing-economy contexts.
Exposure	Agricultural commodity prices, food prices, export-crop shocks, or closely related economic shocks that plausibly affect rural household welfare.	Macro-financial or policy shocks unrelated to commodity markets or food affordability.
Outcomes	Adolescent pregnancy, contraceptive access, fertility, maternal or child health, nutrition, schooling, birth outcomes, or gendered pathways into reproductive risk.	Outcomes with no plausible connection to reproductive health or adolescent vulnerability.
Design and source	Peer-reviewed empirical studies and systematic reviews with abstracts available for screening.	Duplicates, records without abstracts, methods papers, commentary without evidence, or papers with weak conceptual fit.
Final prioritization	Studies offering the strongest leverage for one of three domains: commodity shocks and fertility; commodity shocks and child or adolescent health pathways; adolescent SRH determinants or interventions.	Redundant studies or papers that remained too indirect to support the focused synthesis.

Note. Direct adolescent-specific causal studies were scarce, so adjacent evidence on fertility, nutrition, schooling, maternal or child health, birth outcomes, and service access was retained only when it clarified pathways into adolescent reproductive vulnerability. Developing-economy transferability was judged by the presence of common structural features, including commodity exposure, high food-expenditure burdens, rural service gaps, limited social protection, and gendered barriers to adolescent autonomy.

## Data Availability

No new data were created or analyzed in this study.
